# Transgenic overexpression of furin increases epileptic susceptibility

**DOI:** 10.1038/s41419-018-1076-x

**Published:** 2018-10-17

**Authors:** Yi Yang, Miaoqing He, Xin Tian, Yi Guo, Feng Liu, Yun Li, Haiqing Zhang, Xi Lu, Demei Xu, Ruijiao Zhou, Yuanlin Ma, Wei Wang, Guojun Chen, Yida Hu, Xuefeng Wang

**Affiliations:** 1 Center for Brain Disorders Research, Capital Medical University, 10 Xi tou tiao, You an men, Feng tai District, 100069 Beijing, China; 2 Beijing Institute for Brain Disorders, 10 Xi tou tiao. You an men, Feng tai District, 100069 Beijing, China; 3 Department of Neurology, The First Affiliated Hospital of Chongqing Medical University, Chongqing Key Laboratory of Neurology, 1 Youyi Road, 400016 Chongqing, China

## Abstract

The proprotein convertase Furin plays crucial roles in the pathology of many diseases. However, the specific role of furin in epilepsy remains unclear. In our study, furin protein was increased in the temporal neocortex of epileptic patients and in the hippocampus and cortex of epileptic mice. The furin transgenic (TG) mice showed increased susceptibility to epilepsy and heightened epileptic activity compared with wild-type (WT) mice. Conversely, lentivirus-mediated knockdown of furin restrained epileptic activity. Using whole-cell patch clamp, furin knockdown and overexpression influenced neuronal inhibitory by regulating postsynaptic gamma-aminobutyric acid A receptor (GABA_A_R)-mediated synaptic transmission. Importantly, furin influenced the expression of GABA_A_R β2/3 membrane and total protein in epileptic mice by changing transcription level of GABA_A_R β2/3, not the protein degradation. These results reveal that furin may regulate GABA_A_R-mediated inhibitory synaptic transmission by altering the transcription of GABA_A_R β2/3 subunits in epilepsy; this finding could provide new insight into epilepsy prevention and treatment.

## Introduction

The epilepsies are a heterogeneous group of diseases characterized by recurrent seizures, affecting 1% of people worldwide across different ages and background^1^. Epileptic seizures are caused by abnormal, excessive, and rapid electrochemical discharges by neurons in the brain. The formation of a discharge network of thousands of neurons depends on synaptic transmission. When any dysfunction occurs in adaptable and flexible synaptic transmission, the equilibrium between excitation and inhibition in neuronal networks is disrupted, leading to inappropriate neuronal firing and ultimately the generation of spontaneous, recurrent seizures^2^. Studying some key factors which can effect synaptic signal transmission may provide new ideas for the development of novel methods of epilepsy prevention and treatment.

Furin (paired basic amino acid cleaving enzyme) is a calcium-dependent protease that is expressed ubiquitously in multiple organizations^3^. Furin has a strong preference for basic amino acids and processes substrates at the consensus cleavage site R-X-K/R-R↓, wherein X represents any amino acid but cysteine and is usually not proline^4^. Furin plays crucial roles in a variety of physiological processes and is involved in the pathology of many diseases, such as endocrinopathies^5^, Alzheimer’s disease^6^ and cancer^7^. Furin is involved in the process of cleavage a variety of protein precursors to mature proteins, such as brain-derived neurotrophic factor (BDNF)^8^, which can promote neuronal survival and regulate synapse formation and synaptic plasticity^9^. Moreover, furin has been reported to be the principal endoprotease that cleaves pro-β-nerve growth factor (pro-β-NGF) to β-NGF^10^. Surprisingly, the furin-catalyzed processing of pro-β-NGF controls whether the neurotrophin activates cell-survival or cell-death pathways within innervating neurons^11^. Additionally, furin is a key regulator of the Notch way^12^, which may increase susceptibility to epilepsy development and promote seizure activity in temporal lobe epilepsy (TLE)^13^. Therefore, we hypothesized that furin might have a role in the neuronal synaptic function observed in epilepsy and might thus affect epileptic seizures.

Drug treatment is the main treatment for epileptic patients. However, approximately 20–40% of epileptic patients develop refractory epilepsy, failing to respond to anti-epileptic drugs (AEDs)^14^. TLE is the most common type of refractory epilepsy^15^. In the current study, we discovered that furin was primarily expressed in neurons and that its protein levels were elevated in brain tissues from patients with TLE and from epileptic mice. Subsequently, we generated transgenic mice that overexpress furin in the brain, from which we found that furin has an impact on epileptic activity as monitored by behavioral observations and electroencephalograms (EEGs) in two different epilepsy models. In further analysis, we examined the effect of furin on epileptic electrophysiology using the patch clamp technique, whereby we found that furin affected the inhibitory postsynaptic current (IPSC) in epilepsy. Western blot (WB) and real-time reverse transcription polymerase chain reaction (RT-PCR) results showed that furin affected the transcription of GABA_A_R β2/3 subunits in epilepsy. Taken together, furin affects the function of inhibitory synapses in epilepsy by regulating the transcription of GABA_A_R β2/3 subunits.

## Results

### Expression and cellular localization of furin in brain tissues from patients with TLE and epileptic mice

**Fig. 1:**
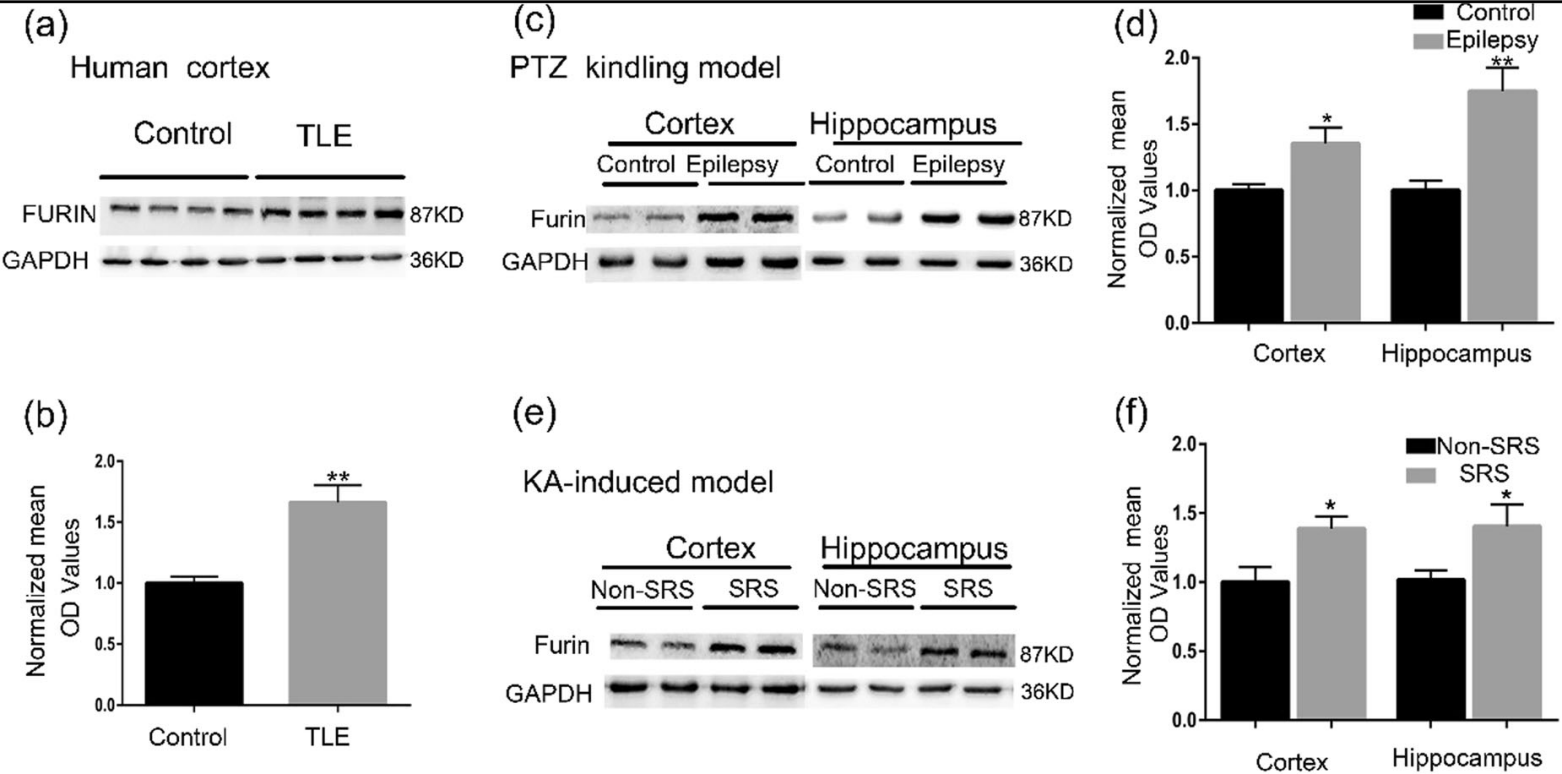
Expression of furin in brain tissues from patients with TLE and from epileptic mice. **a**, **b** Western blots demonstrated that furin protein levels were increased in the temporal neocortex of TLE patients (*n* = 20) compared with that of control patients (*n* = 10). In the PTZ-kindled epilepsy model (**c**, **d**) and the KA-induced epilepsy model (**e**, **f**) furin protein was expressed at higher levels in the cortex and hippocampus of epileptic mice than in those of control mice (*n* = 8 in each group,*P < 0.05, **P < 0.01). Means ± S.E.M. Student’s *t*-tests were performed

To demonstrate the relationship between furin and epilepsy, we tested the expression levels of furin protein in the surgical samples from intractable TLE patients and controls. The level of furin protein in the temporal neocortex dramatically increased by 1.66-fold in patients with epilepsy compared with the control group (**P < 0.01, Fig. 1a, b). Then, we established two chronic epilepsy models of mouse to exclude the possibility that altered furin expression may arise from AEDs in TLE patients. In the pentylenetetrazol (PTZ)- kindled model, the expression of furin in epileptic mice increased significantly by 1.75-fold and 1.35-fold in the hippocampus and temporal cortex, respectively, compared with those of the control group (*n* = 8 in each group,*P < 0.05, **P < 0.01; Fig. 1c, d). In the kainic acid (KA)-induced model, the expression of furin in mice with spontaneous recurrent seizures (SRSs) increased significantly by 1.40-fold and 1.38-fold in the hippocampus and temporal cortex, respectively, compared with those of the non-SRS group (*n* = 8 in each group, *P < 0.05; Fig. 1e, f). These results show that elevated furin expression may be involved in epileptic seizures.

**Fig. 2:**
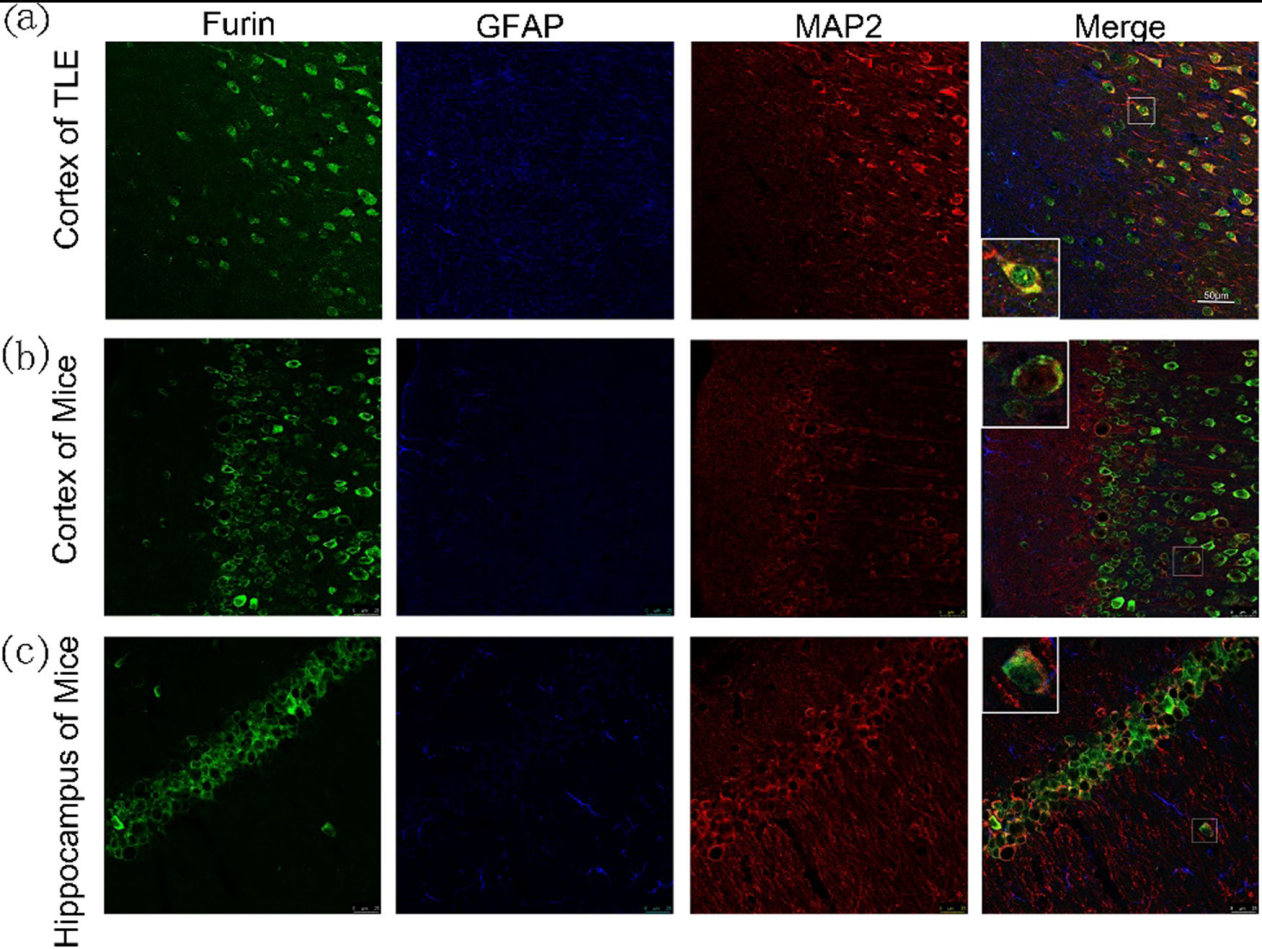
Cellular localization of furin in brain tissues from patients with TLE and from epileptic mice. **a** Immunofluorescence staining demonstrated that FURIN co-localized with the neuronal dendrite marker MAP2, while co-localization of FURIN and the glial cell marker GFAP was not detected in the cortex of TLE patients. The temporal cortex **b** and hippocampal CA1 region (**c**) of epileptic mice corroborated these results.Scale bar: 50 μm

An earlier study showed that furin could be expressed in the CNS;^16^ therefore, we measured the localization of furin expression in brain tissues using immunofluorescence staining. In temporal neocortex tissue from TLE patients, we found that Furin (green) was expressed in neurons rather than glia, as shown by co-localization with the dendritic marker MAP2 (red) and the lack of co-expression with the glial protein GFAP (Fig. 2a). In the epileptic mice, Furin was ubiquitously expressed in neurons of the hippocampal CA1 region (Fig. 2c) and the cortex (Fig. 2b), as shown by co-localization with MAP2. Similarly, Furin was not expressed in GFAP-labeled glial cells. Therefore, this protein was primarily expressed in neurons and not glial cells in epilepsy.

### Effect of furin overexpression and inhibition on behavior in two mouse chronic epilepsy models

**Fig. 3:**
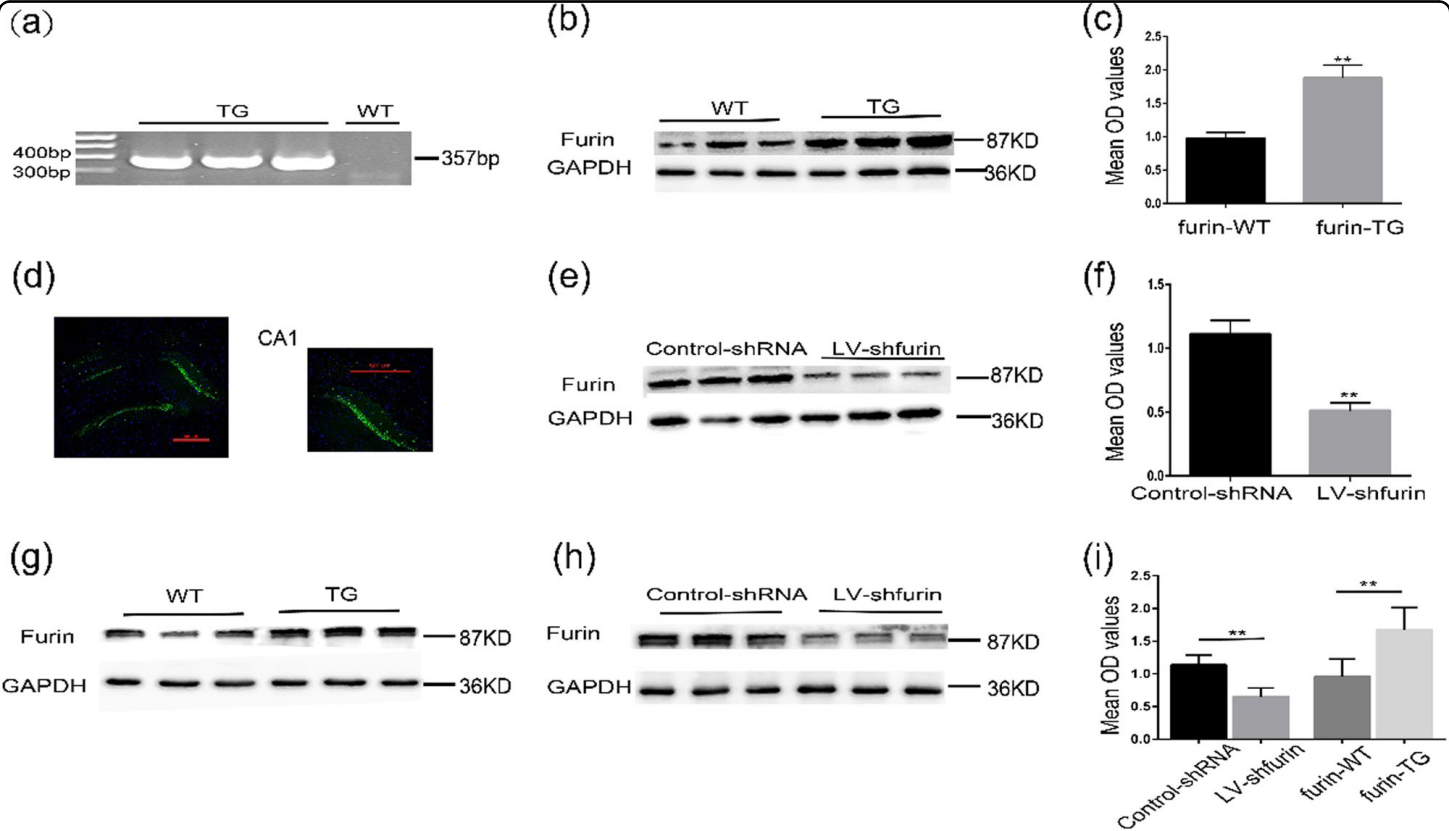
Overexpression and knockdown of furin in mice. **a** Germline transmission of the transgene was verified by PCR analysis of tail DNA using transgene-specific primers. TG mice (357 bp fragment), WT mice (free fragment). **b**, **c** Western blot analysis demonstrated that the protein expression of furin was significantly higher in the TG group than in the WT group (*n* = 5 in each group, **P < 0.01). **d** Fluorescence staining images showing the transfection of LV-shRNA into the mouse hippocampus. Scale bar: 500 μm. **e**, **f** Western blot analysis demonstrated that the expression of furin protein was significantly lower in the LV-sh-furin group than in the control group (*n* = 5 in each group, **P < 0.01). **g–i** Six weeks after the injection of lentiviral vectors, the level of furin protein in the hippocampus was analyzed by Western blot (*n* = 6 in each group, **P < 0.01). Means ± S.E.M. Student’s *t*-tests were performed

**Fig. 4:**
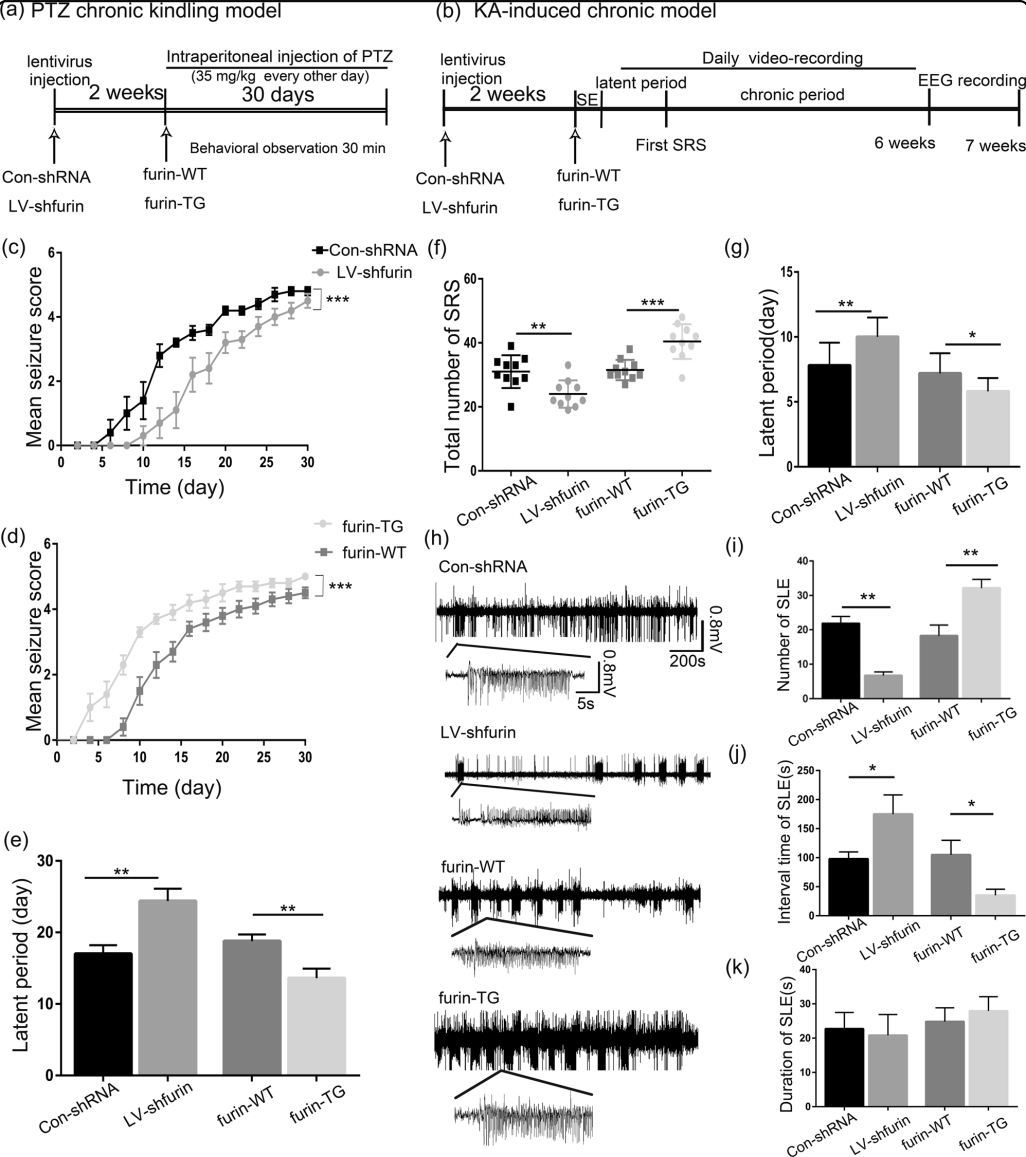
Effects of furin overexpression and inhibition on behavior in chronic epilepsy models. **a**, **b** The concrete steps of the behavioral experiment in the PTZ-kindled chronic epilepsy model and the KA-induced chronic epilepsy model. **c**, **d** Mean seizure scores of PTZ-kindled Con-shRNA, LV-sh-furin, WT, and TG mice over the course of the experiment. **c** Reduced seizure scores were noted in LV-sh-furin mice (*F* = 96.963, ***P < 0.001). **d** Increased seizure scores were noted in furin TG mice (*F* = 117.444, ***P < 0.001) (*n* = 10 in each group). Repeated measures ANOVA was performed. **e** The latency of epileptic seizures was longer in the LV-sh-furin group than in the Con-shRNA group and significantly shorter in the TG group than in the WT group (*n* = 9 or *n* = 10 in each group, **P < 0.01). **f**, **g** In the KA-induced epilepsy model, TG mice showed more frequent SRSs than WT mice, and the latency of SRSs was significantly shorter in the epileptic model than in the WT mice. By contrast, mice injected with LV-sh-furin showed less frequent SRSs than the Con-shRNA group, and the latency of SRSs was significantly longer in TG mice than in Con-shRNA mice (*n* = 9 or *n* = 10 in each group,*P < 0.05, ***P < 0.001). **h** Representative traces of EEG in vivo multichannel electrophysiological recordings from each group. **i** The TG mice showed greater numbers of SLEs than the WT mice, and **j** the interval time between SLEs was shorter in TG mice than in WT mice. The LV-sh-furin group and the Con-shRNA group showed opposite results (*n* = 7 in each group, *P < 0.05, **P < 0.01). **k** There was no significant difference in SLE duration between the TG and WT mice, and the LV-sh-furin and Con-shRNA groups showed the same result (*P* > 0.05). Student’s *t*-tests were performed. The data are expressed as Means ± S.E.M

To examine whether overexpression or inhibition of furin may affect epilepsy, we investigated the PTZ-kindled and KA-induced chronic epilepsy models (Fig. 4a, b). First, furin TG mice and WT mice were identified by PCR (Fig. 3a). WB analysis demonstrated that the protein expression of furin in the TG group (1.877 ± 0.1957) was significantly higher than that of the WT group (0.9732 ± 0.09023) (*n* = 5 in each group, **P < 0.01; Fig. 3b, c). Fourteen days after the injection of LV-sh-furin and control-shRNA lentiviral vectors, GFP autofluorescence was detected in the mouse hippocampus, indicating successful lentiviral infection (Fig. 3d). Similarly, the expression of furin protein in LV-sh-furin group (0.5101 ± 0.06388) was significantly lower than the control group (1.110 ± 0.1078) (*n* = 5 in each group, **P < 0.01; Fig. 3e, f). These findings indicated that furin protein was overexpressed or that its expression was inhibited, as appropriate, in the mouse hippocampus. Furthermore, 6 weeks after the injection of lentiviral vectors, the level of furin protein in the hippocampus was analyzed, with the same result (0.9621 ± 0.1088 in the WT group and 1.678 ± 0.1374 in the TG group; 1.135 ± 0.06203 in the Con-shRNA, 0.6528 ± 0.05462 in the LV-sh-furin, *n* = 6 in each group, **P < 0.01; Fig. 3g–i). These findings indicate the stability of the lentiviral vectors and TG mice.

In the PTZ-kindled model (Fig. 4a), mice were treated with repeated and intermittent intraperitoneal administrations of a subconvulsive dose of PTZ for 30 days. Beginning with almost no observable convulsive behavior, the mice exhibited progressively increasing seizure scores over the course of the experiment (Fig. 4c, d). Lower seizure scores were noted in LV-sh-furin mice than control-shRNA mice (repeated measures-ANOVA, *F* = 96.963). Higher seizure scores were noted in furin TG mice than in WT mice (*F* = 117.444) (***P < 0.001, *n* = 10 in each group). In addition, the latency to the first seizure score >3 was longer in the LV-sh-furin group (24.40 ± 1.708) than in the Con-shRNA group (17.00 ± 1.202), and the latency of epileptic seizures in the TG group (13.60 ± 1.327) was significantly shorter than that in the WT group (18.80 ± 0.9043) (**P < 0.01, Fig. 4e). The KA-induced model (Fig. 4b) is generally established within 2–3 weeks after KA injections. In terms of observed behavioral manifestations (*n* = 9 or 10 in each group), TG mice (40.40 ± 1.714) showed more frequent SRSs than WT mice (31.50 ± 1.003) (***P < 0.001, Fig. 4f), and the latency of SRSs (5.800 ± 0.3266) in the epileptic model was significantly shorter than that in the WT mice (7.200 ± 0.4899) (*P < 0.05, Fig. 4g). By contrast, mice with injected with LV-sh-furin (24.00 ± 1.350) showed less frequent SRSs than the Con-shRNA group (31.00 ± 1.619) (**P < 0.01, Fig. 4f), and the latency of SRSs was significantly longer in LV-sh-furin group (10.00 ± 0.4714) than in Con-shRNA mice (7.800 ± 0.5538) (**P < 0.01, Fig. 4g). In conclusion, furin inhibition suppresses epileptic seizure activity and severity, whereas furin overexpression has the opposite effect.

EEG is an important means of diagnosing epilepsy by recording abnormal discharge of brain neurons. Local field potentials (LFPs) in the mouse hippocampus were recorded in vivo using a multichannel electrophysiological recorder to assess abnormal discharges (*n* = 7 in each group, Fig. 4h). The TG mice (32.14 ± 2.50) exhibited more seizure-like events (SLEs) than the WT mice (18.29 ± 3.099) (**P < 0.01, Fig. 4i), and the interval time between SLEs was shorter in TG mice (35.16 ± 10.52) than in WT mice (104.9 ± 25.00) (*P < 0.05, Fig. 4j). The LV-sh-furin group and the Con-shRNA group showed opposite results (*P < 0.05, **P < 0.01, Fig. 4i, j). There were no significant differences in SLE duration between the TG and WT mice, and the LV-sh-furin and Con-shRNA groups showed the same results (*P* > 0.05, Fig. 4k). Thus, the interventions to manipulate furin expression affected the spontaneous rhythmic electrical activity of cerebral neurons, further supporting a relationship between furin and epilepsy.

### Furin affects GABA_A_R-mediated inhibitory synaptic transmission through postsynaptic mechanism in a brain slice model of epilepsy

When the balance of excitement and inhibition in the brain is disturbed, abnormal discharges can result, leading ultimately to epilepsy. Thus, we utilized whole-cell patch clamping to further study the effect of furin on neuronal excitatory and inhibitory synaptic transmission in a Mg^2+^-free cell model of epilepsy.

**Fig. 5:**
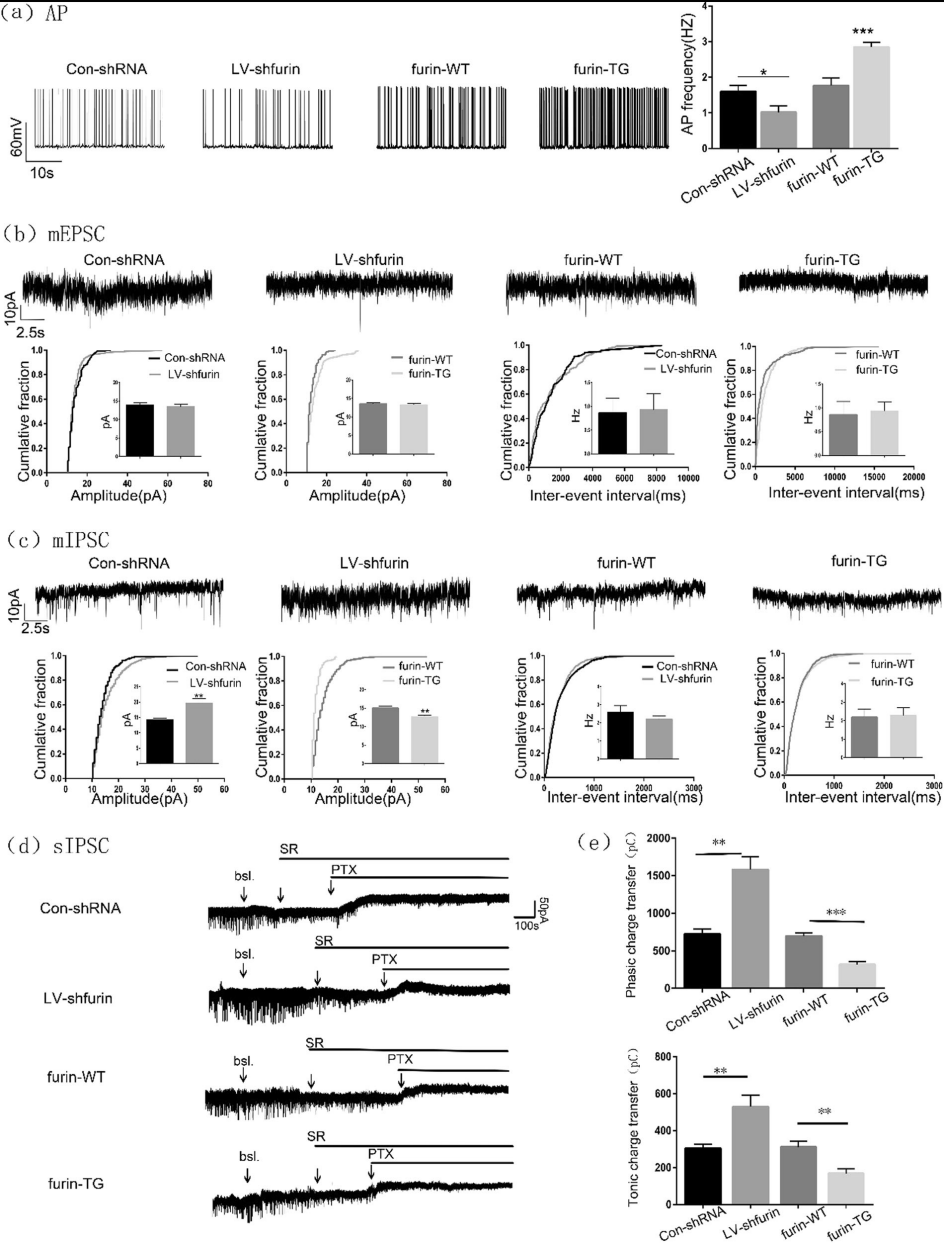
Results of patch clamp recording. **a** Representative traces of APs in the hippocampal CA1 region in each group. The TG mice showed an increase in AP frequency compared with WT mice, while LV-sh-furin infection resulted in a decrease in AP frequency compared with Con-shRNA (*n* = 6 in each group, *P < 0.05, ***P < 0.001). **b** Representative traces of mEPSCs in the hippocampal CA1 region of each group. There was no difference in the amplitude or frequency of mEPSCs between the TG or LV-sh-furin group and the corresponding control group (*n* = 6 or *n* = 8 in each group, *P* > 0.05). **c** Representative traces of mIPSCs in the hippocampal CA1 region of each group. The mIPSCs amplitude was significantly decreased in the TG group but remarkably increased in the LV-sh-furin group (*n* = 7 in each group, **P < 0.01).There was no difference in the frequency of mIPSCs between these 2 groups (*n* = 6 or *n* = 8 in each group, *P* > 0.05). **d** Representative traces of sIPSCs in the hippocampal CA1 region of each group. All traces marked “+SR” showed the changes of phasic charge transfer and traces marked “+PTX” showed the changes of tonic charge transfer. **e** The amount of phasic charge transfer was significantly decreased in the TG group but remarkably increased in the LV-sh-furin group. The amount of tonic charge transfer has the same changes (*n* = 6 in each group, **P < 0.01). Student’s *t*-tests were performed. The data are expressed as Means ± S.E.M

We first examined the action potentials (APs) induced in pyramidal neurons in the hippocampal CA1 area by Mg^2+^-free artificial cerebrospinal fluid (ACSF). The TG mice (2.844 ± 0.1416) showed an increase in AP frequency compared with the WT mice (1.765 ± 0.2098), while LV-sh-furin infection (1.019 ± 0.1744) resulted in a decrease in AP frequency compared with Con-shRNA (1.602 ± 0.1738) (*n* = 6 in each group, *P < 0.05, ***P < 0.001, Fig. 5a).To assess excitatory synaptic transmission, we recorded the miniature excitatory postsynaptic currents (mEPSCs). There was no difference in the amplitude or frequency of mEPSCs between the TG/LV-sh-furin groups and the corresponding control groups (*P* > 0.05, Fig. 5b), thus suggesting that furin do not affect either presynaptic or postsynaptic mechanisms of excitatory transmission in the CA1 neurons. The effects of furin on inhibitory transmission were investigated next. We recorded the miniature IPSCs (mIPSCs) of every group. The mIPSCs amplitude was significantly decreased in the TG group (12.71 ± 0.3842) compared with the WT group (15.03 ± 0.4940), but remarkably increased in the LV-sh-furin group (19.62 ± 1.506) compared with the Con-shRNA group (14.25 ± 0.4324) (*n* = 6 in each group, **P < 0.01, Fig. 5c). However, there was no difference in the frequency of mIPSCs between the TG/LV-sh-furin groups and the corresponding control groups (*P* > 0.05, Fig. 5c).

GABA and glycine are the major inhibitory neurotransmitters in the CNS. Glycine-ergic currents closely resemble GABA-ergic, and GABA_A_R antagonists also act at glycine receptor^17^. Strychnine is an antagonist of glycine that selectively inhibits glycine-ergic activity. In order to determine whether the effect of furin on mIPSCs involves the effect on glycine current, a set of controlled experiments was performed. The results showed that furin overexpression or inhibition did not change either amplitude, frequency or overall charge transfer from the first (no strychnine) and second (with strychnine) experimental epochs observed (*P* > 0.05, Supplementary Fig. 1).Therefore, furin change GABA transmission without affecting glycine-ergic transmission.

GABA_A_-mediated inhibition can occur via synaptic mechanisms or through tonic activation of extrasynaptic receptors. To clarify the effect of furin of synaptic origin, we recorded spontaneous IPSCs. The amount of phasic charge transfer (the changes of area under IPSC curves before and after the SR application) was significantly decreased in the TG group (317.8 ± 41.35pC) compared with the WT group (694.9 ± 40.35pC), but remarkably increased in the LV-sh-furin group (1582 ± 168.6pC) compared with the Con-shRNA group (724.1 ± 69.16Pc) (*n* = 6 in each group, **P < 0.01, ***P < 0.001, Fig. 5e). The tonic charge transfer (area between levels of holding current before and after the PTX application) has the same changes (311.6 ± 30.05pC in the WT group and 168.6 ± 25.08pC in the TG group, 303.4 ± 23.60pC in the Con-shRNA group and 529.4 ± 61.88pC in the LV-sh-furin group; *n* = 6 in each group, **P < 0.01, Fig. 5e). Therefore, we conclude that furin can affect the phasic inhibition mediated by synaptic receptors and tonic inhibition mediated by extrasynaptic receptors.

**Fig. 6:**
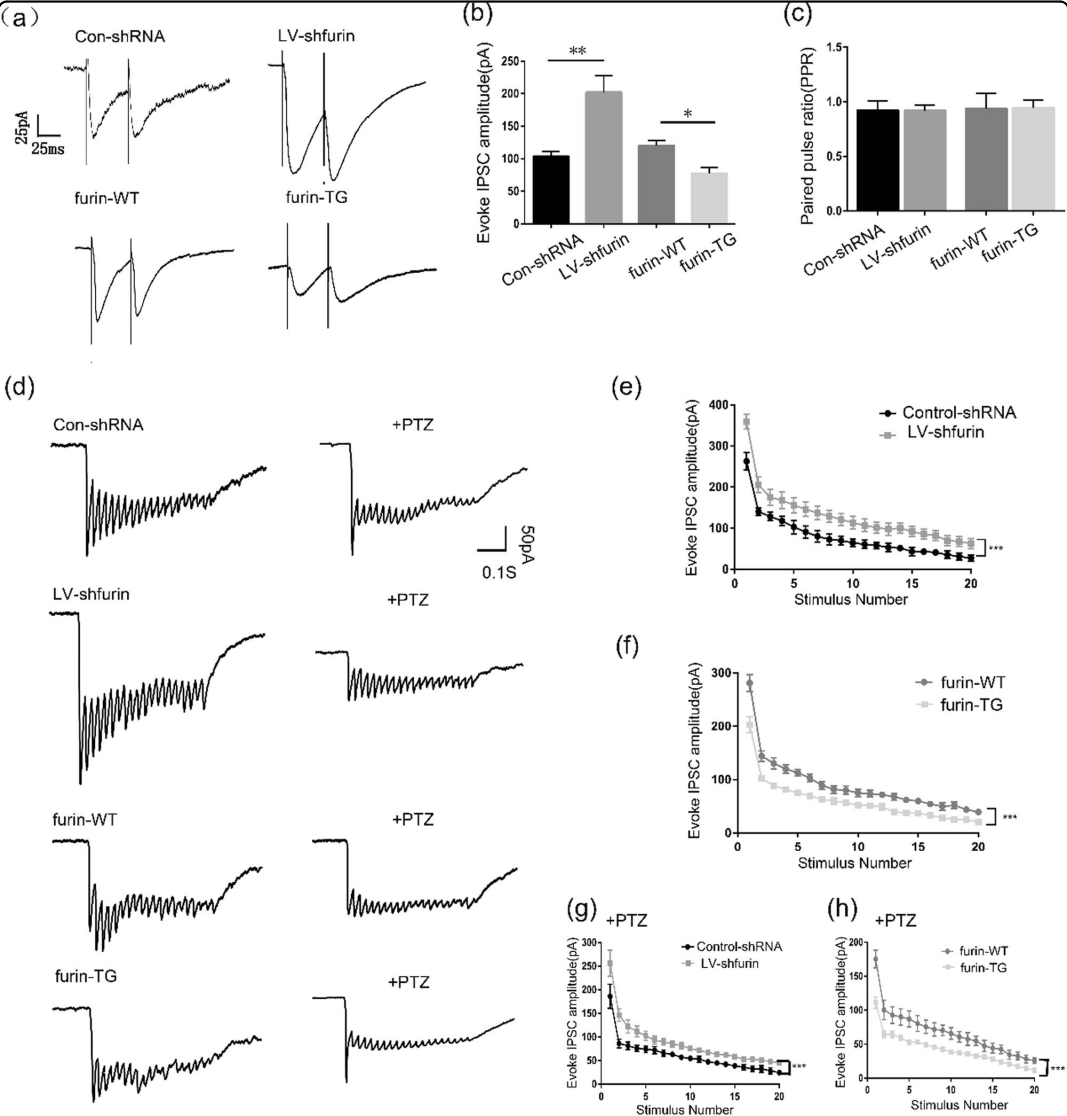
Furin affects GABA transmission through postsynaptic mechanism. **a** Representative traces of the PPR in the hippocampal CA1 region of each group. **b** The first eIPSC amplitude was significantly decreased in the TG group but increased in the LV-sh-furin group (*n* = 6 in each group, *P < 0.05, **P < 0.01). **c** There was no difference in PPR between the TG or LV-sh-furin group and the corresponding control group (*n* = 6 in each group, *P* > 0.05). **d** Representative traces of train evoked IPSCs in the hippocampal CA1 region of each group. The response amplitude decreased with the increase of the number of stimuli. **e** The higher amplitudes were noted in LV-sh-furin group than in the control-shRNA group at every stimulus (*n* = 6 in each group, rm-ANOVA, *F* = 136.901, ***P < 0.001). **f** The lower amplitudes were noted in the TG group than in the WT group at every stimulus (*n* = 6 in each group, *F* = 128.772, ***P < 0.001). **g**, **h** PTZ application resulted in a decrease in amplitude of eIPSC. **g** The higher amplitudes were noted in LV-sh-furin group than in the control-shRNA group at every stimulus (*n* = 6 in each group, *F* = 68.397, ***P < 0.001). **f** The furin-TG group and furin-WT group showed opposite results (*n* = 6 in each group, *F* = 73.596, ***P < 0.001). The data are expressed as Means ± S.E.M

To further clarify whether the effect of furin on inhibitory synapses is generated by presynaptic GABA release mechanisms or postsynaptic GABA_A_Rs, we recorded evoked whole-cell response. Paired-pulse ratios (PPR) were calculated as the ratio between the amplitudes of second evoked IPSP (P_2_) and the first evoked IPSP (P_1_). The P_1_ amplitude was significantly decreased in the TG group (77.34 ± 9.379) compared with the WT group (119.9 ± 8.553), but remarkably increased in the LV-sh-furin group (202.0 ± 26.27) compared with the Con-shRNA group (103.4 ± 7.520) (*n* = 6 in each group, *P < 0.05, **P < 0.01, Fig. 6b). The reduced or increased eIPSC amplitude was not associated with a change in the PPR. A significant difference in the PPR was not observed among the groups (*P* > 0.05, Fig. 6c), which excluded the possibility that furin affects presynaptic inhibitory neurotransmitter release probability. Next, train eIPSCs were recorded. When vesicle release probability approaches physiological maximum, we observed decrease of response amplitude over number of stimulations (Fig. 6d).The results showed that the lower amplitudes were noted in the TG group than in the WT group at every stimulus (rm-ANOVA, *F* = 128.772, ***P < 0.001, Fig. 6f). After perfusion with PTZ, the amplitudes of eIPSCs decreased further due to the partial inhibition of GABA_A_Rs (*F* = 73.596, ***P < 0.001, Fig. 6h). Similarly, the higher amplitudes were noted in LV-sh-furin group than in the control-shRNA group at every stimulus (*F* = 136.901 before PTZ perfusion and *F* = 68.397 after PTZ perfusion, *n* = 6 in each group, ***P < 0.001, Fig. 6e–g). In summary, furin affects GABA transmission through postsynaptic mechanism in epilepsy.

### Furin influences epileptic seizures by changing GABA_A_R β2/3 subunit gene expression

**Fig. 7:**
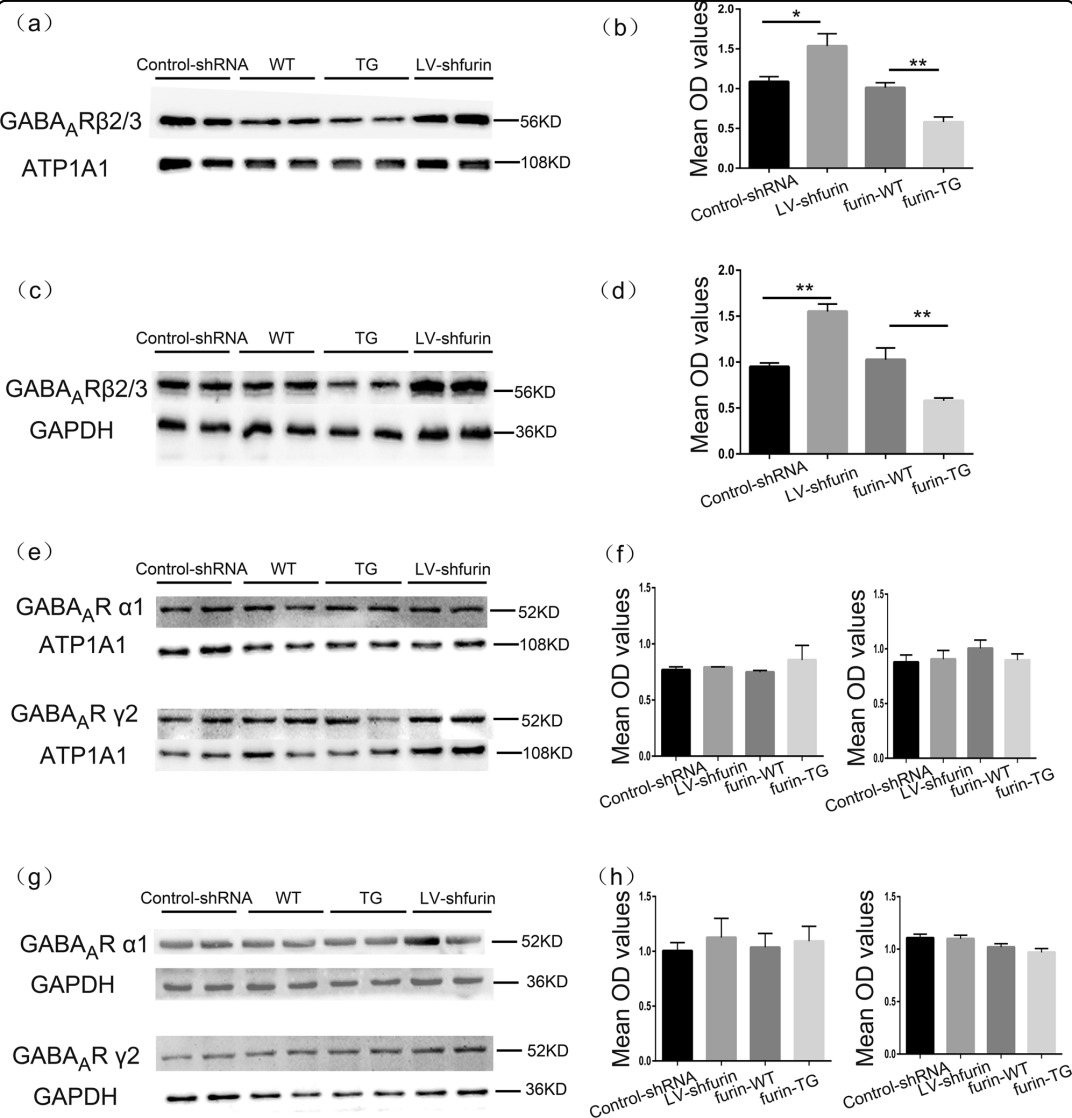
Furin affects the membrane expression and total protein expression of GABAAR β2/3. **a–d** The expression levels of membrane/total GABAAR β2/3 were significantly lower in the TG-furin group and significantly higher in the LV-shRNA group than in the control group (a membrane protein, c total protein) (*n* = 6 in each group, *P < 0.05; **P < 0.01). **e–h** There were no significant differences in the level of total or membrane GABAAR α1 or γ2 between the TG or LV-sh-furin group and the corresponding control group (*n* = 6 in each group, *P* > 0.05). Student’s *t*-tests were performed. The data are expressed as Means ± S.E.M

To investigate the mechanism by which furin affects the inhibitory postsynaptic current in epilepsy, we investigated the GABA_A_R in a KA-induced model. There was no difference in the expression level of total hippocampal GABA_A_R α1/γ2 protein between the TG group and the WT group (*P* > 0.05, Fig. 7f). Similarly, we did not observe a difference in the surface GABA_A_R α1/γ2 levels between the TG group and the WT group (*P* > 0.05, Fig. 7e). The control-shRNA group and the LV-sh-furin group showed the same results (*P* > 0.05, Fig. 7f, e). By contrast, the expression of GABA_A_R β2/3 membrane and total protein was significantly lower in the TG group than in the control WT group, while GABA_A_R β2/3 membrane and total protein expression was significantly higher in the LV-sh-furin group than in the control-shRNA group (*n* = 6 in each group,*P < 0.05; **P < 0.01, Fig. 7a, b).

**Fig. 8:**
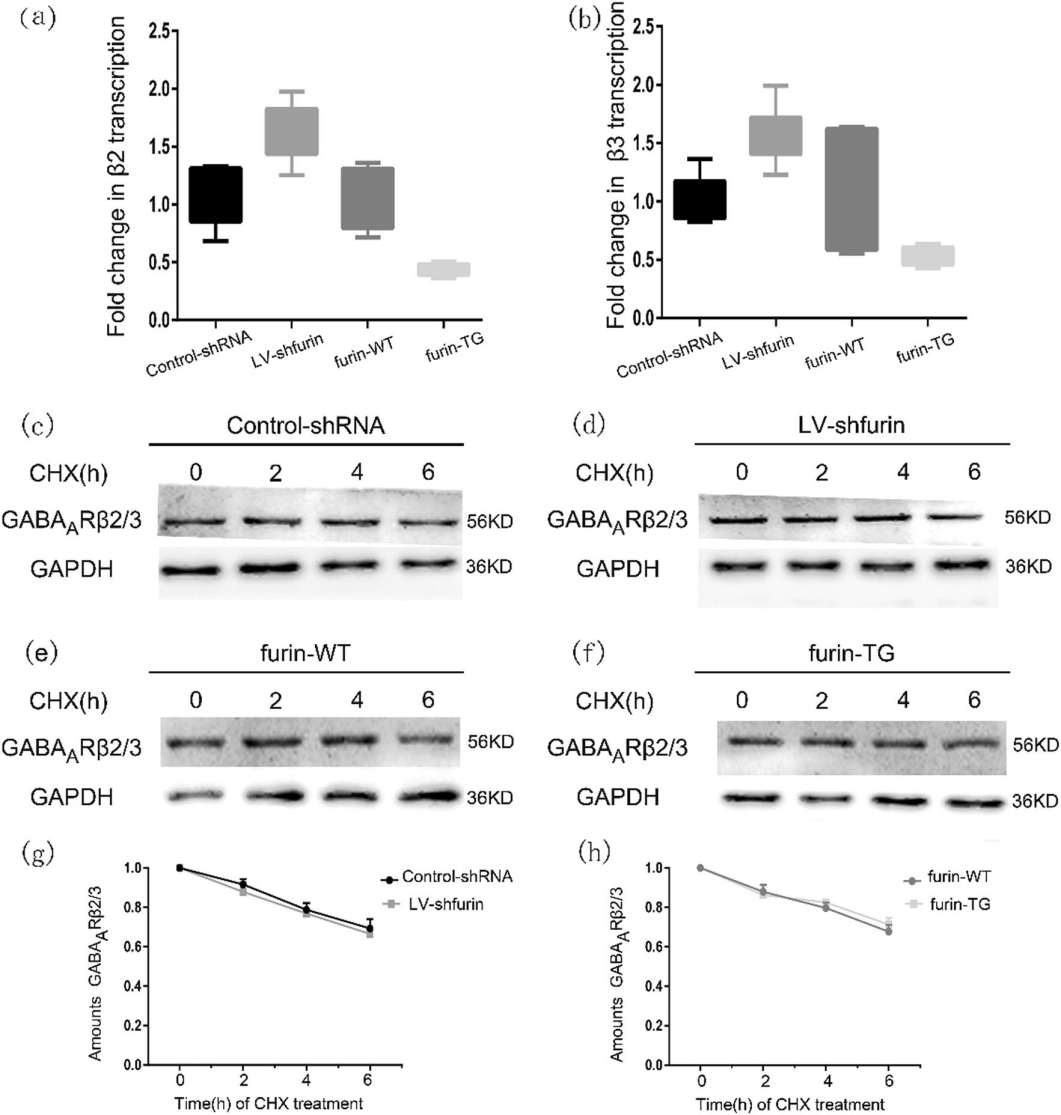
Furin influences GABAAR β2/3 subunit gene expression in epileptic mice. **a**, **b** Amounts of GABAARβ2/3 mRNA in the TG-furin and LV-sh-furin groups relative to the amounts in corresponding control group (*n* = 6 in each group).Fold change was expressed as 2^-ΔΔCt^. **c–h** A CHX-mediated protein degradation assay to estimate the degradation rate of GABAAR β2/3 subunits. After CHX treatment for different lengths of time (0, 2, 4, and 6 h), Western blot analysis was used to measure protein levels. The degradation rates of GABAAR β2/3 proteins in the cell lysates during the CHX treatment did not significantly differ between the TG or LV-sh-furin groups and the corresponding control group (*P* > 0.05). Experiments were conducted in triplicate. The data are expressed as Means ± S.E.M

Changes in protein concentrations depend on the synthesis and degradation of proteins. Therefore, on the one hand, the synthesis of receptor subunits in KA-induced mice was measured. As a result of normalizing the GABA_A_R β2/3 values to GAPDH mRNA (Table 1), the GABA_A_R β2 expression level of the furin-TG group (0.4005 ± 0.03146) was found to be significantly lower than that of the furin-WT group (1.155 ± 0.3236) (*P* = 0.0427). The GABA_A_R β2 expression level of the LV-sh-furin group (1.632 ± 0.1034) was found to be significantly higher than that of the Con-shRNA group (1.026 ± 0.1019) (*P* = 0.0019) (Fig. 8a). We found the same results for GABA_A_R β3 mRNA changes in response to the knockdown and overexpression of Furin (Fig. 8b). On the other hand, we used a cycloheximide (CHX)-mediated protein degradation assay to estimate the degradation rate of GABA_A_R β2/3 subunits. After CHX treatment for different lengths of time (0, 2, 4, and 6 h), cellular protein was extracted from each group and measured (Fig. 8c–f). There were no significant differences in the degradation rates of GABA_A_R β2/3 proteins in the cell lysates during the CHX treatment between the TG/LV-sh-furin groups and the corresponding control groups (Fig. 8g, f).

**Table 1:** Relative quantitation using the comparative Ct method in the experimental and control groups

	GABA_A_Rβ2(2^-△△CT^)	GABA_A_Rβ3(2^-△△CT^)
Control-shRNA	1.026 ± 0.1019	1.016 ± 0.08401
LV-sh-furin	1.632 ± 0.1034	1.577 ± 0.1018
*P*-value	0.0019	0.0017
Furin-WT	1.155 ± 0.3236	1.094 ± 0.1957
Furin-TG	0.4005 ± 0.03146	0.5276 ± 0.03221
*P*-value	0.0427	0.0171

ΔΔ Ct = (Ctgabr2/3-Ct*GAPDH*) _TG_−(Cgabr2/3t-Ct*GAPDH*) _WT_/

ΔΔ Ct = (Ctgabr2/3-Ct*GAPDH*) _Lv-sh-Furin_−(Ctgabr2/3t-Ct*GAPDH*) _Con-shRNA_

## Discussion

Disturbances in synaptic transmission that disrupt the balance between excitatory and inhibitory synapses play a role in the pathogenesis of seizures and epilepsy^2,18^. In the present study, we investigated the role of furin in epilepsy; this enzyme may affect the inhibitory synapses by regulating the transcription of GABA_A_R β2/3 subunits. We first examined the expression and cellular localization of furin in brain tissues from patients with TLE and epileptic mice. Considering ethical limitations, we could not acquire normal brain tissues from humans; therefore, we used histologically normal brain tissue obtained from temporal lobectomies performed to treat head trauma. At the same time, to exclude the possibility that altered Furin expression might arise from AEDs in TLE patients, we investigated Furin expression in mouse models of epilepsy. Using immunofluorescence, Furin is mainly distributed in neurons. Next, to establish that elevated Furin in epilepsy is not merely a concomitant phenomenon, we observed the changes in the behavioral seizures of furin knockdown and furin-overexpressing mice. Two chronic epilepsy model are applied. The PTZ-kindled model has the advantages of progressive pathological response and epileptic activity^19,20^. Hippocampal injection of KA produces strong excitatory effects, resulting in excessive excitation and subsequently inducing epileptic seizures^21^. With a help of transgenic mouse model, we found that TG-furin mice showed heightened epileptic susceptibility, which accelerated the progression of chronic epilepsy and increased the severity of seizures; nevertheless, down-regulation of furin decreased susceptibility to epilepsy. These results suggest that furin is involved in the development of epilepsy.

The abnormal electrical activity in neural networks that characterizes epilepsy is closely related to the balance of excitatory and inhibitory synapses^22^. The processes of synaptic neurotransmission are tightly regulated, including the proteins involved in synaptic vesicle formation, neurotransmitter release and subsequent vesicle endocytosis and recycling; neurotransmitter reuptake; the effects of the neurotransmitter itself on postsynaptic receptors, and subsequent signaling cascades and modulation of the signal communicated through the synapse^23^. Glutamate and GABA are the most important excitatory and inhibitory neurotransmitters, respectively. we used whole-cell patch clamp recording to detect electrophysiological changes at the level of individual neurons. The Mg^2+^-free model is a classic epilepsy model in vitro used to explore the electrophysiological mechanism that induces spontaneous epileptiform discharges^24–27^. In the present study, overexpression of furin increased AP frequency and decreased mIPSC amplitude, with the opposite changes observed upon furin knockdown. At the same time, we have conducted electrophysiological control experiments on brain slices from KA and PTZ models and Mg2+-free model brain slices ((*P* > 0.05, Supplementary Fig. 2). The above results suggest that furin can alter the excitability of neurons and affect inhibitory synaptic transmission in hippocampal slices of epilepsy. Neuronal inhibition can occur via synaptic mechanisms or through tonic activation of extrasynaptic receptors^28^. Apart of classical phasic (synaptic) GABA-ergic signaling, tonic (extrasynaptic) GABA-ergic conductance is an important part of integral inhibitory tone which often plays a pivotal role in interneuronal crosstalk^29^. Overexpression and inhibition of furin induce significant changes of tonic and phasic charge transfer, thus suggesting furin plays an important role on synaptic and extrasynaptic GABA_A_Rs. The mIPSC amplitude might be of both pre- and postsynaptic origin. Presynaptic mechanism: no change release probability in terms of number of synapses activated per certain time interval, but change in a number of vesicle release sites activated per synapse^30^. Postsynaptic mechanism: change opening probability rates and/or number of GABA_A_Rs^23^. Paired and train evoked whole-cell response confirmed furin effect is generated by postsynaptic GABA_A_Rs rather than presynaptic GABA release mechanisms. Deficits in GABA_A_R-mediated neurotransmission are implicated in the etiology of epilepsy^31^. Thus, we speculated that furin might influence neuronal excitability and mIPSCs by regulating postsynaptic GABA_A_Rs in epilepsy.

The subset of GABA_A_Rs found at synapses are composed of two α1, α2, or α3 subunits together with two β2 or β3 subunits and a single γ2 subunit^32^. The number of GABA_A_ receptors in the postsynaptic membrane directly controls the efficacy of GABAergic synaptic transmission^33^. We investigated the expression of the GABA_A_R in an epileptic mouse model. There was no difference in the expression level of total or surface GABA_A_R α1/γ2 protein in the hippocampus between the TG/LV-sh-furin groups and the corresponding control groups. Overexpression of furin reduced the expression of GABA_A_R β2/3 membrane protein, while down-regulation of furin had the opposite effect. It is worth mentioning that total GABA_A_R β2/3 protein displayed the same change whether furin was knocked down or overexpressed in the hippocampus. This finding indicates that the synthesis and/or degradation of GABAAR β2/3 protein may change. To further investigate this mechanism, we used real-time PCR to analyze transcription of mRNA and employed a CHX-mediated protein degradation assay. The results showed that knockdown or overexpression of furin affected the transcription level of GABA_A_R β2/3 but did not affect the degradation.

Furin plays a crucial role as proprotein convertase, whose active substrates such as BDNF are closely related to epilepsy. Studies show increased levels of BDNF in the hippocampus and cerebral cortex of patients with TLE^34,35^. Furin mRNA upregulation appears to be parallel to that of BDNF mRNAs following KA treatment^36^. Signaling by BDNF and its cognate receptor (receptor tropomyosin-related kinase B, TrkB) is critically important for neurogenesis and inhibitory synapse formations^37–39^. At GABAergic synapses, BDNF leads to a rapid and transient increase followed by a lasting reduction in the amplitude of mIPSCs^40,41^. Those evidence support the proposition that furin may affect inhibitory synaptic function.

Collectively, our research reveals a new role of furin in increasing epileptic susceptibility in chronic epilepsy models and emphasizes that furin is part of a molecular mechanism that adjusts the function of the inhibitory synaptic receptor. However, the complex processes regulating synaptic transmission in disease are still incompletely understood. The mechanism whereby furin regulates the GABA_A_ receptor still needs to be explored further and in greater detail.

## Materials and methods

### Human brain tissues

The study protocol was approved by the Institutional Ethics Committee of the First Affiliated Hospital of Chongqing Medical University. All enrolled patients or their family members voluntarily joined this study and gave informed consent.

Twenty patient samples (from 12 males and 8 females), originally acquired at Beijing Tian tan Hospital, Beijing Xuan Wu Hospital, and the First Affiliated Hospital of Chongqing Medical University, China, between 2011 and 2014, were selected randomly from our lab’s established brain tissue bank. The selection was made from patients who had been diagnosed with TLE according to the criteria established by the International League Against Epilepsy^42^. The average patient was 33.00 ± 3.280 years old (range from 9 to 67 years), with a mean disease duration of 11.60 ± 1.857 years (range from 2 to 33 years), and had taken three or more AEDs, followed by surgical resection. The control cases were 10 patients (4 males and 6 females) treated for traumatic brain injury at the First Affiliated Hospital of Chongqing Medical University, China. These patients had histologically normal temporal neocortices, no history of epilepsy or previous AED use, and no other neurological diseases. Their average age was 32.00 ± 3.018 (range from 17 to 51 years). Clinical data for all of the patients are shown in Table 2 and Table 3. There were no significant differences in age or sex distribution between the TLE patients and the controls (*P* > 0.05). All human brain tissues were examined by Western blot.

**Table 2:** Clinical characteristics of TLE patient

Sex (M/F)	Age (years)	Course (years)	History of AEDs	Source of organization	Pathologic diagnosis
M	18	3	VPA/LTG/LEV	LTN	NL, G
M	22	10	CBZ/TPM/PB	LTN	G
F	14	4	LTG/TPM/PB	RTN	NL, G
M	39	15	VPA/TPM/LEV	LTN	NL, G
F	47	20	CBZ/LTG/PHT/PB	LTN	NL, G
F	55	5	PB/OXC/LEV	RTN	G
F	21	3	OXC/LEV/PB	LTN	NL, G
M	17	6	VPA/OXC/LEV	RTN	NL, G
F	9	2	CBZ/PB/LTG	LTN	G
M	33	10	TPM/VPA/LEV/PB	LTN	NL, G
M	28	8	PB/VPA/LEV	RTN	NL, G
M	43	20	CBZ/TPM/LEV/PB	RTN	NL, G
F	36	17	CBZ/LTG/TMP/PB	LTN	NL, G
M	25	5	VPA/LEV/CBZ	LTN	G
F	48	23	CBZ/VPA/LEV	RTN	NL, G
M	33	13	VPA/LEV/PHT	RTN	NL, G
M	27	7	PB/PHT/VPA	LTN	NL, G
F	40	20	CBZ/PB/LTG/LEV	LTN	NL, G
M	38	8	CBZ/VPA/LEV/PB	RTN	NL, G
M	67	33	PHT/TPM/PB	LTN	G

*E* epilepsy, *M* male, *F* female, *AEDs* antiepileptic drugs, *VPA* valproic acid, *LTG* lamotrigine, *LEV* levetiracetam, *CBZ* carbamazepine, *TPM* topiramate, *PB* phenobarbital, *PHT* phenytoin, *OXC* oxcarbazepine, *LTN* left temporal neocortex, *RTN* right temporal neocortex, *NL* neuronal necrosis, *G* glios

**Table 3:** Clinical characteristics of control patients

Patient	Sex (M/F)	Age (years)	Disease diagnosis c	History of AEDs	Source of organization	Pathologic diagnosis
C1	F	34	Brain trauma	None	RTN	Normal
C2	F	40	Brain trauma	None	RTN	Normal
C3	M	26	Brain trauma	None	LTN	Normal
C4	F	34	Brain trauma	None	LTN	Normal
C5	M	28	Brain trauma	None	RTN	Normal
C6	M	37	Brain trauma	None	RTN	Normal
C7	F	17	Brain trauma	None	LTN	Normal
C8	F	23	Brain trauma	None	RTN	Normal
C9	M	51	Brain trauma	None	LTN	Normal
C10	F	30	Brain trauma	None	RTN	Normal

*C* control, *F* female, *M* male, *RTN* right temporal neocortex, *LTN* left temporal neocortex

### Animal experiments

All animal experiments were approved by the Institutional Animal Ethics Committee of the First Affiliated Hospital of Chongqing Medical University, China. In accordance with the NIH Guidelines for the Care and Use of Laboratory Animals, all mice were kept in a specific-pathogen-free (SPF) environment under standard conditions with a 12 h/12 h light/dark cycle, a room temperature of 21–22 °C and a relative humidity of 55 ± 5%, and were provided with sufficient food and water. All mice were obtained from the Experimental Animal Center of Chongqing Medical University.

### Animal model generation and experimental groups

A transgenic mouse line that overexpresses furin in the brain was created on a C57BL/6 J background (CasGene Biotech Co., Ltd, Beijing, China). We constructed a transcription unit by inserting the coding region of the mouse furin cDNA into the MoPrP (mouse prion gene promoter)-polyA cassette and consequently. The linearized MoPrP-furin transcription unit was microinjected into fertilized C57BL/6 J mouse eggs, which were then transferred to the oviducts of pseudopregnant C57BL/6 J females. The transgenic founder mice were obtained and crossed to wild-type (WT) C57BL/6 J mice to establish transgenic lines^43^. Mice were genotyped by PCR analysis of tail DNA using primers specific for an internal fragment of furin. The primer sequences were as follows: 5′-TAGCCAAGCGAAGAGCCAAGAGG-3′ and 5′-GCCATCCAACATCCGCACCC-3′. Furin knockout mice are unable to survive owing to cardiac developmental defects at 10.5 embryonic days^44^. Therefore, lentivirus vectors for furin RNA interference (LV-sh-furin) were constructed according to the manufacturer’s protocol (GeneChem Co., Ltd, Shanghai, China). The sequence of LV-sh-furin was as follows: GAGAATGATGTGGAGATCATCCGTG; the titer of the lentiviral vectors was 3 × 10^8^ TU/ml. Lentivirus vectors coding for GFP only were used as control vectors (Con-shRNA).

The mice were randomly divided into 4 groups: (1) furin-WT mice, (2) furin-TG mice, (3) Control-shRNA mice, and (4) LV-sh-furin mice.

### Mouse models of chronic epilepsy

#### Pentylenetetrazole (PTZ)-kindled epilepsy model

According to the method^45^, PTZ (35 mg/kg) was administered to the mice by intraperitoneal (i.p.) injection every other day for 30 days. After the injections, we observed and recorded the evoked behavioral seizures of the mice for 30 min on the basis of the standard Racine scale:^46,47^ stage I, immobility and staring; stage II, rigid posture; stage III, repetitive movements and head bobbing; stage IV, rearing and myoclonic twitching; V, generalized tonic-clonic seizures with falling. Mice that showed at least three consecutive level 4 or 5 epileptic seizures after receiving PTZ injections were considered completely kindled, and we classified those mice as the epilepsy group. The mice that did not meet the above conditions were regarded as the control group.

#### Kainic acid-induced chronic epilepsy model

According to the method reported^48^, mice were deeply anesthetized and unilaterally injected with 1.0 nmol of KA (Sigma-Aldrich Co., St. Louis, MO, USA) in 50 nl of saline in the right hippocampus. Stereotaxic injections into the dorsal region of the CA1 area were performed at the following coordinates with respect to bregma: anteroposterior, −1.8 mm; mediolateral, −1.5 mm; and 1.5 mm below the dura. We injected KA over a 1-min period with a 0.5-μl microsyringe (Hamilton, Reno, NV, USA). At the end of the injection, the microsyringe remained in situ for an additional 5 min and was finally withdrawn slowly to minimize backflow along the path of the syringe. For 4 weeks, beginning one day after status epilepticus induction, the chronic epilepsy model was confirmed by checking for at least one behavioral SRS with motor manifestations. Only mice with observed SRSs during the chronic phase were included in the epilepsy group, and mice that did not exhibit SRSs were used as controls.

### Behavioral observations

Four groups of male mice weighing 24–28 g per mouse were used to generate two different chronic epilepsy models as described above. In the PTZ-kindled model, behavioral observation was performed for 30 min after each intraperitoneal injection, and the seizures were scored on the five-point scale described above. In the KA-induced epilepsy model, epileptic behaviors were observed and recorded through 24 h of video monitoring after the onset of SE induced by unilateral hippocampal injections of KA^49^.

### In vivo multichannel electrophysiological recording

After the end of the behavioral observation of the KA-induced epilepsy model, LFPs were recorded by implanting a multichannel microwire array (a 4 × 4 array of platinum-iridium alloy wires, each 25 μm in diameter, Plexon, Dallas, TX, USA) in the right dorsal hippocampus (anteroposterior, −1.8 mm; mediolateral, −1.5 mm; and 1.5 mm below the dura). The experimental procedures were as described previously. In accordance with previous research^48^, electrophysiological epileptiform-like discharges were defined as spike activity lasting longer than 5 s with high frequency (>5 Hz) and high amplitude (>2 times the baseline). For each recording session, we determined the frequency and duration of SLEs. Data from 7 different mice in each group were ultimately included in the final statistical analysis.

### Brain slice preparation

Mice weighing 20–30 g were anesthetized with isoflurane and quickly decapitated, and their brain tissues were sliced using a vibratome (Leica, Germany) at a slice thickness of ~300–350 mm. The brain slices were immediately placed in precooled artificial cerebrospinal fluid (ACSF: 125 mM NaCl, 3 mM KCl, 1.25 mM NaH_2_PO_4_, 2 mM MgSO_4_, 2 mM CaCl_2_, 25 mM NaHCO_3_, and 10 mM glucose) and incubated in that solution for 1 h at 35 °C and then at room temperature (22–25 °C). The ACSF was continuously saturated with 95% O_2_ and 5% CO_2_.

### Whole-cell patch clamp recordings

The experimental procedures were performed as described previously^50,51^. We used a convulsant bath solution without MgCl_2_ (Mg^2+^-free ACSF), which can induce spontaneous epileptiform discharges^52^. Firstly, the whole-cell patch-clamp technique was used to measure action potentials (APs) of the pyramidal neurons in the CA1 region at 31 °C using glass pipette electrodes (3–5 MΩ) filled with internal solution (60 mM K_2_SO_4_, 60 mM NMG, 40 mM HEPES, 4 mM MgCl_2_, 0.5 mM BAPTA, 12 mM phosphocreatine, 2 mM Na_2_ATP, and 0.2 mM Na_3_GTP, pH 7.2, adjusted with KOH). To record the mEPSCs, an internal solution (17.5 mM CsCl, 0.5 mM EGTA, 10 mM HEPES, 4 mM ATP, 132.5 mM Cs-gluconate, 5 mM QX-314, PH 7.2) was used. The brain slices were soaked in ACSF containing 1 μM tetrodotoxin (TTX), 10 μM bicuculline and the mEPSCs were recorded when the membrane was voltage-clamped at −70 mV. To record the mIPSCs, an internal solution (100 mM CsCl, 10 mM Hepes, 1 mM MgCl·26H_2_O, 1 mM EGTA, 30 mM NMG, 5 mM MgATP, 0.5 mM Na_3_GDP, and 1 mM creatine phosphate salt) (pH 7.2–7.3) was used. The brain slices were soaked in ACSF containing 20 μM, 6, 7-Dinitroquinoxaline-2, 3(1 H, 4 H)-dione (DNQX), 40 μM D-2-amino-5-phosphonovalerate (APV), 1 μM TTX and the mIPSCs were recorded when the membrane was voltage-clamped at −70 mV. The APs, mEPSCs, mIPSCs in the last 3 min of the recording were used for data analysis. To record the sIPSCs, method same as mIPSC without adding TTX. Spontaneous events were recorded for a 10 min baseline period and for 5–10 min in the presence of SR95531 (0.5 μM) and picrotoxin (10 μM)^53^.The amount of phasic/tonic charge transfer in the 1 min of recording were used for data analysis. Evoked IPSCs were elicited using a monopolar glass stimulating electrode filled with ACSF. To distinguish between pre- and post-synaptic mechanisms, PPR stimulation using the parameters described above and separated by 100 ms was used in this study. Train evoked IPSCs: every 60 sec apply a train of 20 stimuli with interstimulus interval of 50 msec. Then perfusate with low concentration of the low-affinity open channel blocker pentylenetetrazole for 10 min and record eIPSCs. Glycine exclusion experiment: continuous recording mIPSCs, then application of strychnine (0.5 μM)^54^ to cut selectively glycine-ergic activity, then application of picrotoxin (100 μM) to demonstrate absence of activity other than GABAR-delivered.

All recordings were monitored with a Multiclamp 700B amplifier (Axon, USA) to be digitized at 10 kHz and filtered at 2 kHz.The data collected from the pyramidal neurons of 5–8 different mice in each group were ultimately analyzed using Mini Analysis 6.0.1 and Clampfit10.3.

### Real-time reverse transcription polymerase chain reaction (RT-PCR)

Total RNA was isolated from the hippocampus using TRIzol (Takara, Dalian, China) of a 1:20 w/v ratio, according to the manufacturer’s protocol. Next, cDNA was synthesized using HiScript II Q RT SuperMix (Vazyme, Nanjing, China). The gene sequences of the GABAAR subunits were obtained from the National Center for Biotechnology Information (NCBI) database. The primer sequences were as follows:

gabrb2 (mouse): 5′-GCCGTAGGAATGAACATTGATATTGCC-3′ and 5′-GTTGGTCTGCCACTCGGTTGTC-3′;

gabrb3 (mouse): 5′-GCTATGGCTACACTACGGATGACATTG-3′ and

5′-GGCGAAGACAACATTCCTGGAGAC-3′;

GAPDH (mouse):5′-TTGTCATGGGAGTGAACGAGA-3′and 5′-CAGGCAGTTGGTGGTACAGG-3′.

The expression of gabr2/3 and gapdh mRNA was detected by real-time quantitative RT-PCR (q RT-PCR); the reactions were performed on a Bio-Rad iQ5 detection system (Bio-Rad, US) with SYBR Green Master Mix (Takara, Japan). The reaction mixture (20 µl in total) consisted of 10 µl of 2 × SYBR mix, 7.2 µl of nuclease-free water, 0.4 µl of each primer (10 µM) and 2 µl of diluted cDNA. The reaction program was performed using the following steps: 95 °C for 30 s, followed by 40 cycles of 95 °C for 5 s, 60 °C for 10 s, and 72 °C for 15 s. At the end of the cycling process, the temperature was raised from 60 °C to 95 °C to obtain the melting curve. Each reaction was performed in triplicate for each sample. Relative mRNA levels (Δ Ct) were determined by the average cycle threshold value (Ct) followed by normalization to the housekeeping gene GAPDH. The fold changes were quantified by using the 2^-ΔΔCt^ method:^55^

[ΔΔ Ct = (Ctgabr2/3-Ct*GAPDH*) _TG_-(Cgabr2/3t-Ct*GAPDH*) _WT_]/

[ΔΔ Ct = (Ctgabr2/3-Ct*GAPDH*) _Lv-sh-furin_-(Ctgabr2/3t-Ct*GAPDH*) _Con-shRNA_].

### Cell culture and protein degradation assay

Primary hippocampal neurons were isolated from mice during the early postnatal period (p0-p1). After brain extraction, hippocampus isolation, trypsin digestion and filtration, a cell suspension was obtained. The cell suspension was diluted with DMEM (Gibco, US). The final cell concentration was adjusted to 100000/mL along with a final concentration of 20% fetal bovine serum, and the suspension was inoculated into poly-L-lysine-coated 35-mm dish or glass coverslips in six-well plates. Then, the neurons were placed in an incubator at 37℃ and 5% CO_2_. Four hours after being plated, the cells were maintained in Neurobasal medium supplemented with B-27, 0.5mM L-glutamine, 100 U/mL penicillin and 100 μg/mL streptomycin (all from Invitrogen). The neurons were maintained in culture until DIV (day in vitro) 14. CHX was purchased from Sigma-Aldrich. Primary neurons at DIV 14 were treated with 20 μg/ml cycloheximide at 37 °C^56^. At defined time points (0, 2, 4, and 6 h), a radioimmunoprecipitation assay (RIPA) protein extraction kit (Beyotime Institute of Biotechnology, Shanghai, China) was used to extract cellular protein. Western blot was used to analyze the protein degradation.

### Protein extraction and western blot analysis

All mice were killed after the animal experiment was completed. The hippocampal tissues were isolated from the brain and stored in liquid nitrogen. Each sample was processed in duplicate. A RIPA protein extraction kit (Beyotime Biotechnology China) containing phenylmethylsulfonyl fluoride (PMSF; Beyotime Biotechnology China) was used to extract total protein. The Pierce Mem-PER Eukaryotic Membrane Protein Extraction Kit (Pierce, USA) was used for membrane protein extraction. An enhanced bicinchoninic acid (BCA) protein assay kit (Beyotime Institute of Biotechnology) was used to determine protein concentrations. Western blot analysis was performed according to published protocols^57^. SDS-PAGE Sample Loading Buffer-5 × (Beyotime Institute of Biotechnology) was used to denature the proteins. Extracts were resolved by SDS-PAGE (5% spacer gel; 10% separating gel) and then subjected to Western blot analysis. The following primary antibodies were used in this study: rabbit anti-furin (1:1000, Abcam, ab183495), rabbit anti-GAPDH (1:3000, Thermo Scientific, PA1–987), mouse anti-GABAAR β2/3 (1:1000, EMD Millipore, 2860637), rabbit anti-GABRA1 (1:1000, Proteintech, 12410-1-AP), rabbit anti-GABRG2 (1:1000, Proteintech, 14104-1-AP), and horseradish peroxidase (HRP)-conjugated anti-rabbit or anti-mouse secondary antibodies (1:3000,Proteintech). The bands were visualized using WesternBright ECL (Advansta, US) and a Fusion FX5 image analysis system (Vilber Lourmat, France).

### Immunofluorescence labeling

Tissue preparation: Mice were deeply anesthetized and intracardially perfused with 50 ml of 0.9% saline, followed by 50 ml of 4% paraformaldehyde. Then, hippocampal tissues were isolated immediately and stored in 4% paraformaldehyde overnight at 4 °C. The tissues were dehydrated in 20% and then 30% sucrose solution. Finally, samples were cut into 10-μm slices. Immunofluorescence labeling was performed as previously described^58^. First, the tissue sections were deparaffinized and rehydrated before staining. After antigen recovery, sections were permeabilized for 10 min using 0.4% Triton X-100 and blocked using normal goat serum (Zhongshan Golden Bridge, Beijing, China) for 1 h to eliminate nonspecific staining. Then, they were incubated in the primary antibody mixture overnight at 4 °C. On the second day, the sections were incubated with secondary antibodies in the dark for 2 h at 37 °C. Between successive steps throughout the process, the sections were washed using PBS. The primary antibodies included rabbit anti-Furin (1:100,Abcam,ab183495), chicken anti-microtubule-associated protein 2 (MAP2, 1:200,Abcam,ab5392), mouse anti-glial fibrillary acidic protein (GFAP, 1:50,Zhongshan Golden Bridge Inc.; TA500336), DyLight 488-conjugated goat anti-rabbit IgG (1:50, Boster Biological Technology Ltd), DyLight 594-conjugated goat anti-mouse IgG (1:50, Beyotime Institute of Biotechnology), and DyLight 405-conjugated goat anti-chicken IgG (1:50, Beyotime Institute of Biotechnology). Finally, laser scanning confocal microscopy was used to detect immunoreactivity.

### Statistical analysis

All statistical analyses were conducted using the statistical software SPSS 19.0. According to whether the samples exhibited normal distributions and equal variances (determined by the one-sample Kolmogorov–Smirnov test and Levene’s test), the experimental results were statistically assessed using parametric or nonparametric tests. The data from this study are expressed as the Means ± SEM and were analyzed using Student’s *t*-test and repeated measures ANOVA (rm-ANOVA) to determine the significance levels of any differences. Differences were considered significant for *P*-values < 0.05.

## Electronic supplementary material

Supplemental material

Supplementary figure legends
