# Supplementary material for: Transgenic overexpression of furin increases epileptic susceptibility
Source: Cell Death Dis. 2018 Oct 17;9(11):1058. doi: 10.1038/s41419-018-1076-x (PMC6193048; doi:10.1038/s41419-018-1076-x)
Supplement: Supplementary file 1 — Supplemental material [file 41419_2018_1076_MOESM1_ESM.docx]

**Supplementary Fig.1 Furin change GABA transmission without affecting glycine-ergic transmission.**

1. Representative traces of the mIPSC in the hippocampal CA1 region of each group. There was not change either amplitude**(b)**, frequency**(c)** or overall charge transfer **(d)**from the first (no strychnine) and second (with strychnine) experimental epochs between the TG or LV-sh-furin groups and the corresponding control group(n=6 in each group, P>0.05). Student's *t*-tests were performed.

**Supplementary Fig.2 No significant difference between slices in Mg^2+^-free ACSF and slices from KA and PTZ models.**

**(a)**Representative traces of APs in slices in Mg^2+^-free ACSF and slices from KA and PTZ models. **(b)** There was no difference in the AP frequency between three epilepsy models (n=4-5 in each group, P>0.05). **(c)** Representative traces of mIPSCs in the hippocampal CA1 region of each group. There was no difference in the amplitude **(d)** or frequency **(e)** of mIPSCs between three epilepsy models (n=4-5 in each group, P>0.05). ANOVA were performed.
